# Cell mechanics: from single scale-based models to multiscale modelling

**DOI:** 10.1080/17513758.2014.901429

**Published:** 2014-04-01

**Authors:** Christian T.K.-H. Stadtländer

**Affiliations:** ^a^ St. Paul, MN, USA

Biological cells are considered as the building units of life. It was Robert Hooke who coined the word *cell* in histological description of cork and other specimens using coarse optical lenses, as published in his *Micrographia* in 1665 [10]. A few years later, Anthonie van Leeuwenhoek used a self-built microscope to observe living microorganisms which he called *animalcules* (‘little animals’) which are known today as protozoa, bacteria, and other forms of life [6]. Since these early years of observation, researchers have come a long way to decipher many features of life, ranging from the structural and functional organization of single- and multi-cell organisms to entire populations, communities, and ecosystems. Researchers recognized that the mathematical language is also a powerful tool for explaining biological phenomena. More specifically, the dynamics of biological systems can be described, simulated, and often predicted in the form of mathematical formulas, algorithms, and computer models. In recent years, computational biology (which includes a quantitative data analysis approach, theoretical methods, mathematical modelling, and computational simulation techniques) has become essential in biological research. This is because modern experimental methods (e.g. advanced imaging techniques, biochemical, genetic, immunological, and molecular analyses) are generating massive data sets at an unprecedented rate, detail, and complexity.

The book by Chauvière, Preziosi, and Verdier focuses on an area of research known as *cell mechanics*. Here, investigators attempt to illuminate how cells sense and identify, interact with each other, and respond to the physical properties of the environment in which they live. Cell mechanics, which draws investigators from various disciplines such as biology, physics, mathematics, computer science, and engineering, has proven to be indispensable for elucidating mechanisms involved in many aspects of cell dynamics, including cell structure, movement, and adhesion [2–4] embryogenesis and embryonics [7], wound healing and tissue engineering [1,11], viable cell growth arrest and apoptosis [5,8], as well as the development of diseases such as asthma, glaucoma, and cancer [9].

The editors point out in the preface that the book is the result of many years of collaboration among researchers of the European Research and Training Network on a topic entitled ‘Modeling, Mathematical Methods and Computer Simulation of Tumour Growth and Therapy’. The book contains reviews that describe experimental and applied mathematical approaches to illuminate how cells behave at various scales. The editors emphasize that it became clear while researching these biological phenomena from different points of view that ‘multiscale problems are ubiquitous and fundamental in cell mechanics’.

*Cell Mechanics: From Single Scale-Based Models to Multiscale Modelling* is divided into four major parts: ‘From Subcellular to Cellular Properties’ (Part I), ‘Single Cell Migration Modelling’ (Part II), ‘Mechanical Effects of Environment on Cell Behaviour’ (Part III), and ‘From Cellular to Multicellular Models’ (Part IV). These parts contain a total of 15 chapters which have been written by 50 contributors. In addition to the chapters, the preface, and information about the editors and contributors, the book also contains a table of contents and an index which I both tested and found to be functional.

In the first three chapters, which make up Part I, the authors describe issues related to the movement of cells. Chapter 1 introduces the reader to the structural and mechanical properties of the cytoskeletal network which consists of a dynamic assembly of macromolecules forming actin filaments, intermediate filaments, and microtubules, and interacting with or controlled by proteins, cross-linkers, and molecular motors. The authors review data from rheology (stress–strain relationship) studies of single living cells (e.g. myoblasts, epithelial cells, macrophages) at different time and length scales, and present a semi-phenomenological model for cell rheology that can predict the mechanical response of a cell submitted to a controlled stress. In the second chapter, the authors point out that most living cells are able to perform a directed motion, for example, by swimming in a liquid environment, by crawling on a solid support, or by squeezing through a three-dimensional matrix of fibres. They look at the actin-based cell propulsion system and describe the interplay between material properties (the physical mechanisms) and growth processes (i.e. actin polymerization and organization). The third chapter is about cell motility and cancer. The authors describe tumourigenesis as a multistep process and review tumour suppressor genes and the molecular mechanisms involved in cell migration, adhesion, and invasion. They discuss methods for *in vitro* cell migration studies, including two- and three-dimensional migration assays.

Part II (Chapters 4–6) is about the mathematical modelling of single cell migration events. More specifically, the authors of the fourth chapter review the biology of cell polarization and locomotion/migration, events that play a central role in the development and maintenance of tissues in multicellular organisms. They mention that the polarization of a cell defines the direction of migration as well as the cell division axis, and is thus responsible for the three-dimensional structures of tissues, organs, and the whole organism. The authors review various models (e.g. the cellular Potts model and the ‘reactive flow’ model) and then propose a generalized continuum model useful for studying the coupling of cytoplasm (cytoskeletal) and adhesion dynamics. They demonstrate that relatively simple laws for the small-scale mechanics and kinetics of events (e.g. filament polymerization, pushing/sliding, binding/pulling on adhesion sites) can be combined into a nonlinearly coupled system of differential equations which model the polarization and migration behaviour on the large-scale cell level. In the fifth chapter, the authors describe the movement via lamellipodia, which are thin membrane-bound, mostly filamentous actin-containing sheets of cytoplasm that cells protrude at the front end and retract at the rear. They show a multiphase evolution model for lamellipodia with arbitrary shape that allows relating computationally the structure and dynamics of the actin network to the traction forces and shape changes that constitute the fascinating amoeboid movement. The authors of Chapter 6 reiterate that the motility/migration of living cells is a complex and highly integrated process in which cytoskeletal assembly, actin turnover, the contractility of actomyosin, and adhesion dynamics are all closely interlinked and regulated by the properties of the extracellular environment. They present a computational framework (numerical simulations), constructed from an existing mathematical model of fibroblast cell deformations, for the investigation of the coupling of these processes.

The third part contains four chapters in which the authors discuss cell–environment interactions from the mechanical point of view. In Chapter 7, a model is described which demonstrates how a flow field under varying shear stresses can affect the dynamic behaviour of a cell (represented by an adhesive microbead) in close contact with a wall. The authors perform microscale calculations of bond-formation, -advection, and -breakage to predict macroscale cell motion. They identify three different regimes which may overlap for some parameter values: (1) when the sphere adheres to the wall, the bonds prevent both sliding and translational motions; (2) when the sphere tank-treads on the wall, the bonds prevent sliding motion; and (3) when the sphere is free from adhesive forces, most of the bonds are broken. The eighth chapter provides an interesting discussion about our understanding of adhesion sites as mechanosensitive cellular elements. Adhesion sites are considered as ‘clusters of membrane-associated proteins constituting a discrete physiochemical link between the cytoskeleton and the extracellular environment’. The authors discuss the physicochemical mechanism(s) by which cells sense and adapt to the mechanical properties of the extracellular environment.

Chapter 9 is about tumour cell migration. This process, which occurs during the formation of metastases, requires a link between adhesion anchoring and the reorganization of the cytoskeleton. As an experimental model, the authors use migrating T24 cancer cells on polyacrylamide gel substrates with different stiffnesses and discuss the results in light of observations that such cancer cells exert less traction than other cell types. The tenth chapter is entitled ‘Single Cell Imaging of Calcium in Response to Mechanical Stimulation’. The authors discuss the importance of calcium ions (Ca^2+^) in the regulation of many cellular activities and provide design strategies for Ca^2+^ imaging. Furthermore, they describe how the mechanical stimuli or physical forces can be perceived by cells and transduced into biochemical responses – a process known as mechano-transduction.

The final part (Part IV) is entitled ‘From Cellular to Multicellular Models’. The authors of Chapter 11 describe the mathematical framework for modelling the migration of a cell population (i.e. the collective movement of cells) in the extracellular matrix (ECM). They look at the heterogeneity/anisotropy of the ECM medium and examine events such as chemotaxis, haptotaxis, and repellent quorum sensing migration. They explain that cell migration is an essential feature of many physiological and pathological phenomena in biology, such as embryonic development, tissue homeostasis, immune response, and wound healing, as well as metastasis dissemination and tumour invasion. The authors point out that the characteristics of the spreading of a cell population can vary greatly and is depending on both the local micro-environment and the function of the migrating cell(s).

The following three chapters are about mathematical modelling of cell populations as it relates to developmental biology and cancer (Chapter 12), embryogenesis (Chapter 13), and cancer invasion (Chapter 14). In more detail, the authors of the twelfth chapter emphasize that ‘From the earliest embryonic stages to the complexity of the adult, the ability of cell populations to adhere to each other or the surrounding ECM is of critical importance to the survival of the organism’. They demonstrate, for example, the mathematical modelling of cell adhesion, cohesion through adhesion, and cell–cell sorting. In Chapter 13, the authors attempt to quantitatively describe processes in embryo morphogenesis, using a multiscale approach. They believe that ‘Models must span from subcellular mechanisms of cellular mechanics, adhesion, and polarized cell behaviours to a mechanical understanding of substantial portions of the embryo and its environment’. This, again, shows that mathematical modelling is complex and requires knowledge and skills in mathematics, physics, computation, and biology, as well as many adjacent fields. The modelling steps from benign tumour to invasive cancer are laid out in Chapter 14. Here, the authors look at tumour growth and angiogenesis, as well as later stages of cancer, including invasion and intravasation.

The final chapter of Part IV (Chapter 15) is devoted to the description of the Delaunay Object Dynamics (DOD) method. The authors explain that the DOD platform allows investigators to separate the phenotype of a cell seen in experiments (e.g. surface markers, signalling cascades, etc.) from more universal biophysical phenotypes (e.g. surface area, cell division, etc.). This provides the opportunity to uncover subtle effects of the biophysical cell features on tissue organization. The DOD is essentially a tool which can be used to examine the behaviour of a large number of highly motile cells in certain tissues, each having its own phenotype dynamics and mechanical properties. The authors illustrate this method with a simulation of fast-migrating cells in secondary lymphoid tissue.

This book is comprehensive and discusses many issues important in biological dynamics in general and in cell mechanics in particular. The authors demonstrate the great value of mathematical tools for examining biological phenomena at different scales. What I like most about this book is the range of topics selected by the editors and discussed in a remarkable way by the groups of authors who contributed to the individual chapters. I also like that each part of the book contains a brief summary of the contents of all associated chapters. Based on my observations as a book reviewer, it is rare to find such an approach, but it is an excellent idea.

The reader will find the numerous illustrations useful as they sufficiently support the information provided in the text. I will describe two specific examples: The first example is Figure P1 (Preface, p. x) which illustrates the multiscale view of cell mechanics. This schematic describes the subcellular scale, cell scale, and macroscopic scale (biological tissue). I believe this schematic is well placed and useful as it allows the reader to envision the different levels of investigation and modelling. The second example is Figure 6.1 (p. 161). This figure shows three interacting components that contribute to cell motility: adhesion, cytoskeleton, and extracellular environment. Inserting this figure in the text helps the reader understand the complexity of cell motility and the various biological components that need to be (are) considered for biological investigation and for constructing a computational framework.

I noticed that few illustrations are presented in colour but are somewhat oddly placed without legends in the middle of Chapter 14 (between pp. 398 and 399). A specific example is the set of three images depicting the adhesion of T24 cells to substrates with various stiffnesses. This figure is shown in Chapter 9 in black-and-white (Figure 9.10, p. 259) and then again in colour in Chapter 14. I believe showing this figure once in colour with legend in Chapter 9 would have been a better choice. The reference sections are placed at the end of each chapter and contain between 23 and 81 entries, which should be sufficient for readers who want to access additional information.

In conclusion, I believe that Chauvière, Preziosi, and Verdier have edited a useful text for the instructor, student, and researcher interested in cell mechanics and mathematical modelling. The book provides considerable value with comprehensive reviews of biological and mathematical concepts, methodologies, and applications. I recommend *Cell Mechanics: From Single Scale-Based Models to Multiscale Modelling* to the reader who wishes to gain an insight into this fascinating field.
